# Increased Serum Angiotensin II Is a Risk Factor of Nonalcoholic Fatty Liver Disease: A Prospective Pilot Study

**DOI:** 10.1155/2019/5647161

**Published:** 2019-11-13

**Authors:** Yue Li, Feng Xiong, Wen Xu, Side Liu

**Affiliations:** ^1^Guangdong Provincial Key Laboratory of Gastroenterology, Department of Gastroenterology, Nanfang Hospital, Southern Medical University, Guangzhou, China; ^2^Department of Gastroenterology, Shenzhen Hospital, Southern Medical University, Shenzhen, China

## Abstract

**Background and Aims:**

Nonalcoholic fatty liver disease (NAFLD) is one of the most prevalent chronic liver diseases. In this prospective study, we aim to explore the role of angiotensin II (Ang II) and NLRP3 inflammasome in NAFLD patients.

**Methods:**

We prospectively enrolled 96 patients in our hospital from September 2014 to February 2016. Patients were divided into two groups (NAFLD group and Control group), and the serum Ang II level, IL-1*β*, IL-18, and lipids were analyzed. Correlation and multivariable analyses were used in order to identify the potential risk factors of NAFLD.

**Results:**

Although the two groups share a similar demographic background, the Ang II level of NAFLD group patients was significantly higher than that of the Control group (42.18 ± 12.37 vs. 36.69 ± 13.90, *p* = 0.014) when abdominal ultrasound was used for grouping. This finding was confirmed when a FibroScan Cap value was selected to divide participants into the NAFLD group and Control group (41.16 ± 13.06 vs. 34.85 ± 12.64, *p* = 0.040). Multivariable analysis showed that Ang II level is an independent risk factor of NAFLD whether abdominal ultrasound (OR = 1.056, *p* = 0.037) or FibroScan Cap value (OR = 1.069, *p* = 0.013) was deemed as the diagnostic standard. Furthermore, stepwise regression analysis was carried out between Ang II with other parameters and we discovered that Ang II had a linear correlation with IL-1*β*.

**Conclusion:**

Ang II levels of NAFLD patients significantly increased, and elevated Ang II level is an independent risk factor of NAFLD. Our preliminary results also indicate that Ang II may promote the development of NAFLD by activating NLRP3 inflammasome.

## 1. Introduction

Nonalcoholic fatty liver disease (NAFLD) has become one of the most common chronic liver diseases. NAFLD affects over 30% of the US adult population, reaching levels as high as 75-100% in obese individuals [1]. Statistics from the National Health and Nutrition Examination Survey showed that 34% of American adults were obese [2]. Furthermore, it is estimated that more and more people worldwide will be overweight or obese [3], which is closely associated with a number of metabolic syndrome including NAFLD and type II diabetes. NAFLD is now recognized as a pathological spectrum of disease, ranging from simple hepatic steatosis to nonalcoholic steatohepatitis (NASH), fibrosis, and cirrhosis [4]. As it is well known, a two-hit hypothesis is one of the most important mechanisms explaining the development and progression of NAFLD/NASH [5]. Recently, NLRP3 inflammasome has drawn considerable attention. Multiple studies have demonstrated that inflammasome activation promoted inflammation, triglyceride accumulation, and insulin resistance in NASH [6–11]. It is generally recognized that inflammasome activation is the result of two distinct signals: one that activates the transcription of prointerleukin-1*β* (IL-1*β*) and another that mediates the assembly of the inflammasome [12, 13]. Mitochondrial dysfunction mediated NLRP3 inflammasome activation via increased mitochondrial reactive oxygen species, and oxidized mitochondrial DNA is an important pathway of the second signals [14–17]. Ang II is considered the ultimate effector of the renin-angiotensin system (RAS) on systemic blood pressure regulation and has been implicated as a major contributor in NAFLD development and progression in recent years [18]. Furthermore, Ang II blocking drugs, such as angiotensin-converting enzyme inhibitors (ACEIs) and angiotensin receptor blockers (ARBs), have been also found to reduce the development of NAFLD in large amounts of literature [18–23]. However, regulatory mechanism underlying these effects of Ang II blocking drugs on NAFLD remains to be unsolved and the relationship between Ang II and NLRP3 inflammasome also has not been reported. In this study, we aimed to determine whether Ang II and NLRP3 inflammasome are involved in NAFLD patients. We also aim to determine the possible mechanism underpinning the effect of Ang II on NAFLD patients based on our clinical data.

## 2. Patients and Methods

A total of 121 adult patients in our hospital were prospectively collected during the period from September 2014 to February 2016. The inclusion criteria included (1) 18 to 70-year-old adults and (2) no serious accompanied diseases such as advanced malignant tumor and organ failure. The exclusion criteria included (1) the administration of angiotensin II receptor blockers, *β* receptor antagonist, vasodilator, diuretics, and other drugs that have effects on angiotensin levels; (2) virus, alcohol, drugs, and other coexisting causes of chronic liver disease; (3) infectious diseases and autoimmune diseases that may have effects on IL-1*β* and IL-18 levels; (4) patients with mental disorders; and (5) pregnant women and nursing mothers. However, 96 patients fulfilled our inclusion and exclusion criteria and were eventually enrolled in the current study. Blood samples were taken from NAFLD patients (NAFLD group) and healthy volunteers (Control group). Body weight, BMI, alanine aminotransferase, lipids, fasting glucose, fasting insulin, homeostasis model assessment insulin resistance (HOMA-IR), plasma renin activity (PRA), plasma Ang II level, plasma aldosterone, and NLRP3 inflammasome downstream cytokines IL-1*β* and IL-18 were collected and analyzed. The diagnosis of NAFLD required that [24] (1) there was hepatic steatosis by imaging or histology; (2) there was no significant alcohol consumption (>20 g/day for women and >30 g/day for men); (3) there were no competing etiologies for hepatic steatosis; and (4) there were no coexisting causes for chronic liver disease. Abdominal ultrasound and FibroScan examination were selected as the imaging evaluation to detect the presence of hepatic steatosis, due to the fact that most NAFLD patients were simple hepatic steatosis, making it difficult to obtain liver biopsy. Two experienced operators with more than 5000 cases of ultrasound examination performed the abdominal ultrasound, and they were trained to detect steatosis according to the same criteria before the study. Abdominal ultrasound examination for the diagnosis of NAFLD required at least two of the following three criteria [25, 26]: (1) in the diffused enhancement of the near-field echo of the liver, the echo was stronger than that of the kidney; (2) the intrahepatic duct structure display was not clear; and (3) the far-field echo of the liver was fading. FibroScan examination is a noninvasive, immediate, objective, and efficient method to detect and quantify steatosis [27], which was approved by the Food and Drug Administration for the noninvasive assessment of liver disease in 2013. Optimal cut-offs of the FibroScan Cap value have been recommended as 238 dB/m for the detection of steatosis [28]. In our study, all the FibroScan examinations were carried out by one certified operator using a FibroScan device (FibroScan 502F01269, Echosens, Paris, France). Blood chemistry and lipid profiles were measured using a blood chemistry analyzer (Olympus AU600, Tokyo, Japan). HOMA-IR was calculated using the equation homeostasis model assessment: insulin resistance = [fasting insulin (mU/mL) × fasting glucose (mmol/L)]/22.5. Serum IL-1*β* and IL-18 levels in the supernatants were measured with a commercial ELISA kit (RayBiotech, Inc., Atlanta, America) according to the manufacturer's protocols. This research was approved by the Institutional Review Board of Nanfang Hospital affiliated to Southern Medical University (Ethical No. NFEC-2014-086), and all participants provided written informed consent.

### 2.1. Statistical Analysis

All data are expressed as the means ± SEM. The difference of measurement data between the groups was detected by the analysis of Student's *t*-test or Wilcoxon test. Correlation analysis was used to explore the relationship between Ang II, IL-1*β*, IL-18, and NAFLD. Two variables in accordance with normal distribution were analyzed using Pearson correlation analysis, and the variables that do not conform to the normal distribution or classification variables were analyzed using the Spearman correlation analysis. Multivariate analysis was used to find the risk factors of NAFLD, and receiver operating curve (ROC) analysis was also performed. A value of *p* < 0.05 was considered to be statistically significant.

## 3. Results

### 3.1. Ang II Levels of NAFLD Patients Significantly Increased, and Elevated Ang II Level Is an Independent Risk Factor of NAFLD

**Figure 1:**
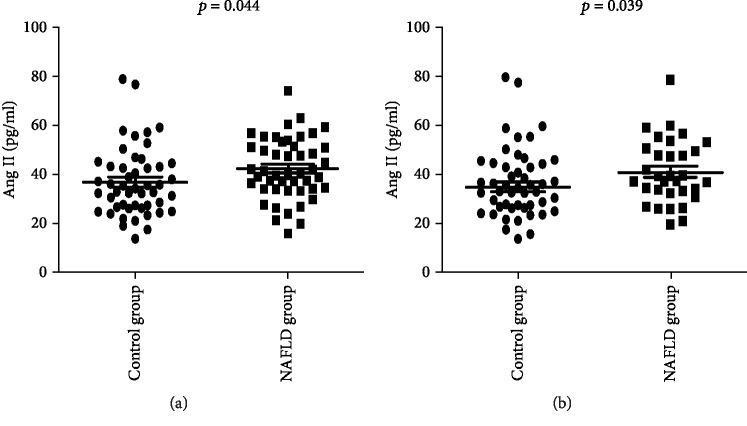
Ang II levels of NAFLD patients significantly increased. (a) The Ang II level of the NAFLD group was significantly higher than that of the Control group according to abdominal ultrasound results (NAFLD group (42.18 ± 12.37) vs. Control group (36.69 ± 13.90); *p* = 0.014). (b) We also confirmed the same finding when the patients were divided into two groups according to the FibroScan Cap value.

**Figure 2:**
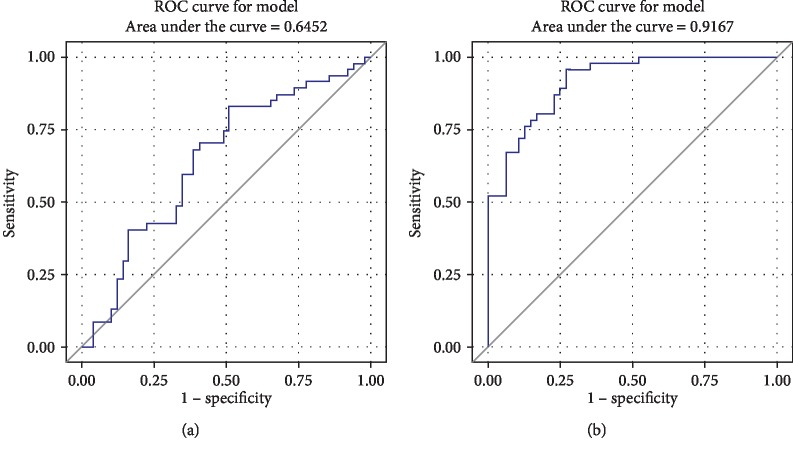
Receiver operating curve (ROC) analysis showed that Ang II alone can discriminate control and NAFLD patients where the area under curve (AUC) was 0.6452 (a) and, especially when Ang II was combined with body weight, TG, and serum insulin which are shown in Table 2, the AUC was 0.9167 (b), suggesting that Ang II was an important factor associated with NAFLD.

Firstly, the patients were divided into two groups (NAFLD group and Control group) according to the abdominal ultrasonography results. The demographic data of the NAFLD group (male/female, 40/7; age, 47.49 ± 6.86 years) and Control group (male/female, 33/16; age, 48.53 ± 10.65 years) were similar (Table 1). However, significant differences were observed between the two groups concerning about body weight, BMI, alanine aminotransferase (ALT), triglycerides, high-density lipoprotein cholesterol (HDL), low-density lipoprotein cholesterol (LDL), serum insulin, HOMA-IR, Ang II, IL-1*β*, and IL-18. The Ang II level of the NAFLD group was significantly higher than that of the Control group (NAFLD group (42.18 ± 12.37) vs. Control group (36.69 ± 13.90), *p* = 0.014) (Figure 1). Multivariate logistic analysis showed that the risk factors of NAFLD included weight (OR = 1.126, *p* = 0.001), triglycerides (OR = 2.289, *p* = 0.010), serum insulin (OR = 1.279, *p* = 0.014), and Ang II level (OR = 1.056, *p* = 0.037) when abdominal ultrasound results were selected as the measuring standard (Table 2). Furthermore, ROC analysis showed that Ang II alone can discriminate control and NAFLD patients with an area under curve (AUC) of 0.6452. Especially when Ang II was combined with body weight, TG, and serum insulin which were risk factors shown in Table 2, the AUC was 0.9167 (Figure 2).

Then, the patients were divided into two groups according to the FibroScan Cap value and we also confirmed that the Ang II level of patients with FibroScan Cap values greater than 238 was significantly higher than that of patients with FibroScan Cap values less than 238. (41.16 ± 13.06 vs. 34.85 ± 12.64, *p* = 0.040) (Table 3). Multivariate analysis showed that risk factors of NAFLD included body weight (OR = 1.118, *p* = 0.001), serum albumin (OR = 1.306, *p* = 0.019), and Ang II level (OR = 1.069, *p* = 0.013) when FibroScan Cap values were selected as the measuring standard (Table 4). We found that Ang II level is always an independent risk factor of NAFLD, whether abdominal ultrasound results or FibroScan Cap value was selected as the measuring standard. Ang II may play an important role in the pathogenesis of the disease.

### 3.2. NLRP3 Inflammasome Activation and the Release of Its Downstream Inflammatory Cytokines Were Involved in the NAFLD/NASH

Correlation analysis showed that FibroScan Cap values had significant correlation with IL-18 and IL-1*β*. The correlation coefficient between the Cap value and IL-18 is 0.254 (*p* = 0.029). The correlation coefficient between the Cap value and IL-1*β* is 0.377 (*p* = 0.011). Two variables in accordance with normal distribution were analyzed using Pearson correlation analysis. The variables that do not conform to the normal distribution or classification variables were analyzed using the Spearman correlation analysis.

### 3.3. Ang II May Promote Liver Inflammation and the Progression of NAFLD/NASH by Activating NLRP3 Inflammasome and Its Downstream Inflammatory Cytokines

When we carried out correlation analysis between Ang II and the other observational index, we also discovered that Ang II level had significant correlation with serum insulin, aldosterone, and IL-1*β*. The Pearson correlation coefficient between Ang II and IL-1*β* is 0.366 (*p* = 0.005). Furthermore, stepwise regression revealed that Ang II only had significant linear correlation with IL-1*β*, and we deduced the following linear regression equation: Ang II = 1.648 IL − 1*β* + 35.924 (*p* = 0.013).

## 4. Discussion

Ang II blockade drugs ACEIs and ARBs have been universally accepted as the first-line drugs for the treatment of hypertension based on the Joint National Committee on prevention, detection, evaluation, and treatment of high blood pressure guidelines [29]. Experimental studies have also demonstrated the beneficial effects of ACEIs and ARBs on the development and progression of NAFLD [18–21, 30–33]. Additionally, a prospective clinical study with small sample size suggests that treatment with losartan results in the improvement of serum liver enzyme levels and hepatic necroinflammation [23]. In a larger study, 54 patients with NASH and hypertension were randomly assigned either to the valsartan group (standard dose 80 mg/d, *n* = 26), r to the telmisartan group (standard dose 20 mg/d, *n* = 28). Both ARBs reduced transaminase levels and improved insulin resistance, but telmisartan showed a higher efficacy regarding insulin resistance and histology [34]. However, the mechanism behind the effects of Ang II on NAFLD remained unclear. Our data demonstrated that there are significantly increased Ang II levels in patients with NAFLD. Ang II levels of NAFLD patients significantly increased, and this may be the theoretical basis for the therapeutical effect of Ang II blocking drugs for NAFLD. We also found that Ang II level was one independent risk factor of NAFLD patients, whether abdominal ultrasound results or FibroScan Cap value was selected as the diagnostic standard, indicating that Ang II may contribute to the progression of NAFLD. ROC analysis showed that Ang II can predict NAFLD and, when Ang II was combined with body weight, TG, and serum insulin which were all risk factors of NAFLD, the AUC was 0.9167, also suggesting that Ang II was an important factor associated with NAFLD.

Numerous researches have showed that the activation of NLRP3 inflammasome and the release of downstream IL-1*β* and IL-18 can promote the process of liver inflammation, triglyceride deposition, and insulin resistance during the NAFLD/NASH [6–11, 17]. In the present study, it is noted that the FibroScan Cap value had a significant correlation with IL-1*β* and IL-18. Thus, it can be seen that Ang II and NLRP3 inflammasome-mediated cytokines IL-1*β* and IL-18 are all involved in NAFLD patients. However, the relationship between Ang II and NLRP3 inflammasome has not been reported, and the interacting mechanism remains unclear. In our previous experimental study, we have found that Ang II can activate the NLRP3 inflammasome in hepatocyte and induce caspase-1-dependent cell apoptosis. Meanwhile, in a methionine-choline-deficient diet-induced mouse NASH model, Ang II blocking drugs losartan and perindopril can downregulate expression of NLRP3-related protein and alleviate the liver steatosis. On the basis of the previous work, we try to explore the relationship between Ang II and NLRP3 inflammasome from the aspects of clinical data for the first time. Our results revealed that Ang II had a significant linear correlation with IL-1*β* and the equation of linear regression was Ang II = 1.648 IL − 1*β* + 35.924 (*p* = 0.013). This shows that Ang II may promote liver inflammation and the progression of NAFLD/NASH by activating NLRP3 inflammasome and its downstream inflammatory cytokines.

One of the major limitations is the limited sample size, which included 96 patients, thus limiting the power to detect significant differences of some parameters. In addition, this study is a prospective observational study, lacking intervention measures, and the persuasive power is relatively limited. Therefore, randomized controlled clinical trials and interventional studies are needed to evaluate the specific mechanism of the Ang II effect on NAFLD patients.

## 5. Conclusion

Ang II levels of NAFLD patients significantly increased, and the elevated Ang II level is an independent risk factor of NAFLD. Our preliminary findings also indicate that Ang II may promote liver inflammation and the progression of NAFLD/NASH by activating NLRP3 inflammasome and its downstream inflammatory cytokines.

## Figures and Tables

**Table 1:** Clinical and laboratory characteristics of the study patients divided into two groups according to abdominal ultrasound results. Significant *p* values are in bold.

Variables	Control group (*N* = 49)	NAFLD group (*N* = 47)	*p* value
Age (years)	48.53 ± 10.65	47.49 ± 6.86	0.572
Gender (male, %)	33, 67.35%	40, 85.11%	0.056
Body weight (kg)	63.95 ± 10.07	76.37 ± 9.47	**<0.001**
BMI value	22.97 ± 2.81	26.21 ± 2.47	**<0.001**
ALT (U/L)	19.85 ± 8.56	27.02 ± 11.81	**0.001**
AST (U/L)	21.83 ± 7.06	23.36 ± 6.34	0.270
Albumin (g/L)	40.68 ± 2.57	41.73 ± 4.65	0.171
CRP (mg/L)	2.62 ± 6.23	1.22 ± 0.99	0.131
TG (mmol/L)	1.30 ± 0.62	2.36 ± 1.71	**<0.001**
CHOL (mmol/L)	5.14 ± 1.04	5.23 ± 1.15	0.702
HDL (mmol/L)	1.39 ± 0.86	1.01 ± 0.23	**0.005**
LDL (mmol/L)	3.24 ± 0.84	3.30 ± 0.89	0.747
VLDL (mmol/L)	0.62 ± 0.31	0.93 ± 0.48	**<0.001**
FBG (mmol/L)	5.11 ± 0.83	5.48 ± 1.06	0.061
Serum insulin (mIU/L)	5.29 ± 2.80	9.19 ± 4.26	**<0.001**
HOMA-IR	1.23 ± 0.83	2.26 ± 1.34	**<0.001**
Ang II (pg/mL)	36.69 ± 13.90	42.18 ± 12.37	**0.014**
PRA (pg/mL)	0.50 ± 0.62	0.95 ± 3.00	0.311
ALD (pg/mL)	17.03 ± 5.94	19.31 ± 8.95	0.142
PRA/ALD	127.10 ± 206.73	106.60 ± 129.15	0.565
IL-18 (pg/mL)	629.26 ± 259.00	743.54 ± 290.51	**0.045**
IL-1*β* (pg/mL)	1.81 ± 1.56	2.36 ± 3.85	**0.049**

**Table 2:** Multivariate analysis showed the risk factors of NAFLD when abdominal ultrasound results were selected as measuring standard.

Variables	*B*	SE	Wald	*p* value	OR value	95% CI of OR value	
						Upper	Lower
Body weight	0.118	0.035	11.621	**0.001**	1.126	1.052	1.205
TG	0.828	0.319	6.718	**0.010**	2.289	1.224	4.281
Serum insulin	0.246	0.100	6.049	**0.014**	1.279	1.051	1.555
Ang II	0.054	0.026	4.344	**0.037**	1.056	1.003	1.111

**Table 3:** Clinical and laboratory characteristics of the study patients divided into two groups according to the FibroScan Cap value.

Variables	FibroScan Cap value < 238 (*N* = 42)	FibroScan Cap value > 238 (*N* = 32)	*p* value
Age (years)	48.17 ± 9.42	48.50 ± 7.94	0.872
Gender (male, %)	33, 78.57%	28, 87.50%	0.371
Body weight (kg)	66.44 ± 11.19	76.41 ± 9.34	**<0.001**
BMI value	23.72 ± 3.13	25.81 ± 2.59	**0.003**
ALT (U/L)	22.00 ± 11.10	26.78 ± 11.39	0.074
AST (U/L)	22.31 ± 7.14	23.81 ± 7.18	0.374
Albumin (g/L)	40.93 ± 2.47	42.26 ± 2.86	**0.036**
CRP (mg/L)	2.75 ± 6.72	1.19 ± 1.01	0.198
TG (mmol/L)	1.72 ± 1.46	2.09 ± 1.62	0.314
CHOL (mmol/L)	5.15 ± 0.97	5.44 ± 1.08	0.230
HDL (mmol/L)	1.35 ± 0.92	1.06 ± 0.22	0.094
LDL (mmol/L)	3.20 ± 0.81	3.52 ± 0.84	0.107
VLDL (mmol/L)	0.75 ± 0.44	0.84 ± 0.47	0.377
FBG (mmol/L)	5.14 ± 0.86	5.47 ± 1.18	0.165
Serum insulin (mIU/L)	6.06 ± 3.27	8.65 ± 4.57	**0.007**
HOMA-IR	1.44 ± 0.95	2.13 ± 1.46	**0.016**
Ang II (pg/mL)	34.85 ± 12.64	41.16 ± 13.06	**0.040**
PRA (pg/mL)	0.55 ± 0.60	1.00 ± 3.60	0.427
ALD (pg/mL)	17.53 ± 5.67	19.29 ± 9.17	0.312
PRA/ALD	101.74 ± 176.88	122.85 ± 134.82	0.577
IL-18 (pg/mL)	678.69 ± 319.41	741.79 ± 222.06	0.343
IL-1*β* (pg/mL)	1.47 ± 1.26	1.82 ± 1.12	0.339

**Table 4:** Multivariate analysis showed the risk factors of NAFLD when the FibroScan Cap value was selected as the measuring standard.

Variables	*B*	SE	Wald	*p* value	OR value	95% CI of OR value	
						Upper	Lower
Body weight	0.112	0.034	11.086	**0.001**	1.118	1.047	1.194
Albumin	0.267	0.114	5.505	**0.019**	1.306	1.045	1.632
Ang II	0.066	0.027	6.151	**0.013**	1.069	1.014	1.126

## Data Availability

Data are available in the database of Nanfang Hospital and in the Clinical Study Office of the Department of Gastroenterology.

## Abbreviations

NAFLD: Nonalcoholic fatty liver disease
NASH: Nonalcoholic steatohepatitis
Ang II: Angiotensin II
RAS: Renin-angiotensin system
ACEIs: Angiotensin-converting enzyme inhibitors
ARBs: Angiotensin receptor blockers
CRP: C-reactive protein
LDL: Low-density lipoprotein
HOMA-IR: Homeostasis model assessment of insulin resistance
ALT: Alanine aminotransferase
AST: Aspartate aminotransferase
BMI: Body mass index
TG: Triglyceride
FBG: Fasting blood glucose
HbA1c: Glycosylated hemoglobin.

## Supplementary Materials

Supplementary Materials We performed the Hosmer-Lemeshow test and provided a calibration plot for the logistic model to confirm that our logistic model in Table 2 is reliable. We also performed the Hosmer-Lemeshow test and provided a calibration plot for the logistic model to confirm that our logistic model in Table 4 is reliable.

## References

[1] Browning J. D., Szczepaniak L. S., Dobbins R. (2004). Prevalence of hepatic steatosis in an urban population in the United States: impact of ethnicity. *Hepatology*.

[2] Flegal K. M., Carroll M. D., Ogden C. L., Curtin L. R. (2010). Prevalence and trends in obesity among US adults, 1999-2008. *JAMA*.

[3] Harford K. A., Reynolds C. M., McGillicuddy F. C., Roche H. M. (2011). Fats, inflammation and insulin resistance: insights to the role of macrophage and T-cell accumulation in adipose tissue. *The Proceedings of the Nutrition Society*.

[4] Cohen J. C., Horton J. D., Hobbs H. H. (2011). Human fatty liver disease: old questions and new insights. *Science*.

[5] Day C. P., James O. F. W. (1998). Steatohepatitis: A tale of two "hits"?. *Gastroenterology*.

[6] Vandanmagsar B., Youm Y. H., Ravussin A. (2011). The NLRP3 inflammasome instigates obesity-induced inflammation and insulin resistance. *Nature Medicine*.

[7] Stienstra R., van Diepen J. A., Tack C. J. (2011). Inflammasome is a central player in the induction of obesity and insulin resistance. *Proceedings of the National Academy of Sciences of the United States of America*.

[8] Lamkanfi M., Kanneganti T. D. (2012). The inflammasome: a remote control for metabolic syndrome. *Cell Research*.

[9] Dixon L. J., Flask C. A., Papouchado B. G., Feldstein A. E., Nagy L. E. (2013). Caspase-1 as a central regulator of high fat diet-induced non-alcoholic steatohepatitis. *PLoS One*.

[10] Wree A., Eguchi A., McGeough M. D. (2014). NLRP3 inflammasome activation results in hepatocyte pyroptosis, liver inflammation, and fibrosis in mice. *Hepatology*.

[11] Szabo G., Csak T. (2012). Inflammasomes in liver diseases. *Journal of Hepatology*.

[12] Martinon F., Burns K., Tschopp J. (2002). The inflammasome: a molecular platform triggering activation of inflammatory caspases and processing of proIL-*β*. *Molecular Cell*.

[13] Bauernfeind F. G., Horvath G., Stutz A. (2009). Cutting edge: NF-*κ*B activating pattern recognition and cytokine receptors license NLRP3 inflammasome activation by regulating NLRP3 expression. *The Journal of Immunology*.

[14] Zhou R., Yazdi A. S., Menu P., Tschopp J. (2011). A role for mitochondria in NLRP3 inflammasome activation. *Nature*.

[15] Tschopp J., Schroder K. (2010). NLRP3 inflammasome activation: the convergence of multiple signalling pathways on ROS production?. *Nature Reviews Immunology*.

[16] Nakahira K., Haspel J. A., Rathinam V. A. (2011). Autophagy proteins regulate innate immune responses by inhibiting the release of mitochondrial DNA mediated by the NALP3 inflammasome. *Nature Immunology*.

[17] Kubes P., Mehal W. Z. (2012). Sterile inflammation in the liver. *Gastroenterology*.

[18] Matthew Morris E., Fletcher J. A., Thyfault J. P., Rector R. S. (2013). The role of angiotensin II in nonalcoholic steatohepatitis. *Molecular and Cellular Endocrinology*.

[19] Hirose A., Ono M., Saibara T. (2007). Angiotensin II type 1 receptor blocker inhibits fibrosis in rat nonalcoholic steatohepatitis. *Hepatology*.

[20] Kudo H., Yata Y., Takahara T. (2009). Telmisartan attenuates progression of steatohepatitis in mice: role of hepatic macrophage infiltration and effects on adipose tissue. *Liver International*.

[21] Rong X., Li Y., Ebihara K. (2010). Angiotensin II type 1 receptor-independent beneficial effects of telmisartan on dietary-induced obesity, insulin resistance and fatty liver in mice. *Diabetologia*.

[22] Paschos P., Tziomalos K. (2012). Nonalcoholic fatty liver disease and the renin-angiotensin system: implications for treatment. *World Journal of Hepatology*.

[23] Yokohama S., Yoneda M., Haneda M. (2004). Therapeutic efficacy of an angiotensin II receptor antagonist in patients with nonalcoholic steatohepatitis. *Hepatology*.

[24] Chalasani N., Younossi Z., Lavine J. E. (2012). The diagnosis and management of non-alcoholic fatty liver disease: practice guideline by the American Association for the Study of Liver Diseases, American College of Gastroenterology, and the American Gastroenterological Association. *The American Journal of Gastroenterology*.

[25] Farrell G. C., Chitturi S., Lau G. K. K., Sollano J. D., for the Asia–Pacific Working Party on NAFLD1 (2007). Guidelines for the assessment and management of non‐alcoholic fatty liver disease in the Asia–Pacific region: executive summary. *Journal of Gastroenterology and Hepatology*.

[26] Mishra P., Younossi Z. M. (2007). Abdominal ultrasound for diagnosis of nonalcoholic fatty liver disease (NAFLD). *The American Journal of Gastroenterology*.

[27] Tapper E. B., Castera L., Afdhal N. H. (2015). FibroScan (vibration-controlled transient elastography): where does it stand in the United States practice. *Clinical Gastroenterology and Hepatology*.

[28] Sasso M., Beaugrand M., de Ledinghen V. (2010). Controlled attenuation parameter (CAP): a novel VCTE™ guided ultrasonic attenuation measurement for the evaluation of hepatic steatosis: preliminary study and validation in a cohort of patients with chronic liver disease from various causes. *Ultrasound in Medicine & Biology*.

[29] Chobanian A. V., Bakris G. L., Black H. R. (2003). Seventh report of the joint national committee on prevention, detection, evaluation, and treatment of high blood pressure. *Hypertension*.

[30] Wei Y., Clark S. E., Morris E. M. (2008). Angiotensin II-induced non-alcoholic fatty liver disease is mediated by oxidative stress in transgenic TG(mRen2)27(Ren2) rats. *Journal of Hepatology*.

[31] Nabeshima Y., Tazuma S., Kanno K., Hyogo H., Chayama K. (2009). Deletion of angiotensin II type I receptor reduces hepatic steatosis. *Journal of Hepatology*.

[32] Shirai Y., Yoshiji H., Noguchi R. (2013). Cross talk between toll‐like receptor‐4 signaling and angiotensin‐II in liver fibrosis development in the rat model of non‐alcoholic steatohepatitis. *Journal of Gastroenterology and Hepatology*.

[33] Yoshiji H., Noguchi R., Ikenaka Y. (2009). Losartan, an angiotensin-II type 1 receptor blocker, attenuates the liver fibrosis development of non-alcoholic steatohepatitis in the rat. *BMC Research Notes*.

[34] Georgescu E. F., Ionescu R., Niculescu M., Mogoanta L., Vancica L. (2009). Angiotensin-receptor blockers as therapy for mild-to-moderate hypertension-associated non-alcoholic steatohepatitis. *World Journal of Gastroenterology*.

