# Supplementary material for: Increased Serum Angiotensin II Is a Risk Factor of Nonalcoholic Fatty Liver Disease: A Prospective Pilot Study
Source: Gastroenterol Res Pract. 2019 Nov 13;2019:5647161. doi: 10.1155/2019/5647161 (PMC6881577; doi:10.1155/2019/5647161)

**Supplementary Table 2**

| **effect** | **ORu** | **CIu** | **Pvalueu** | **ORm** | **CIm** | **Pvaluem** |
| --- | --- | --- | --- | --- | --- | --- |
| AngII | 1.033 | 1.000~1.067 | 0.048 | 1.057 | 1.004~1.112 | 0.035 |
| Insulin | 1.467 | 1.226~1.755 | <0.001 | 1.289 | 1.063~1.563 | 0.010 |
| TG | 3.459 | 1.744~6.861 | <0.001 | 2.294 | 1.228~4.286 | 0.009 |
| Weight | 1.137 | 1.076~1.202 | <0.001 | 1.126 | 1.052~1.206 | 0.001 |

（1）Hosmer-Lemeshow test：chisq= 10.855, df=8, P= 0.210

（2）Calibration plot:


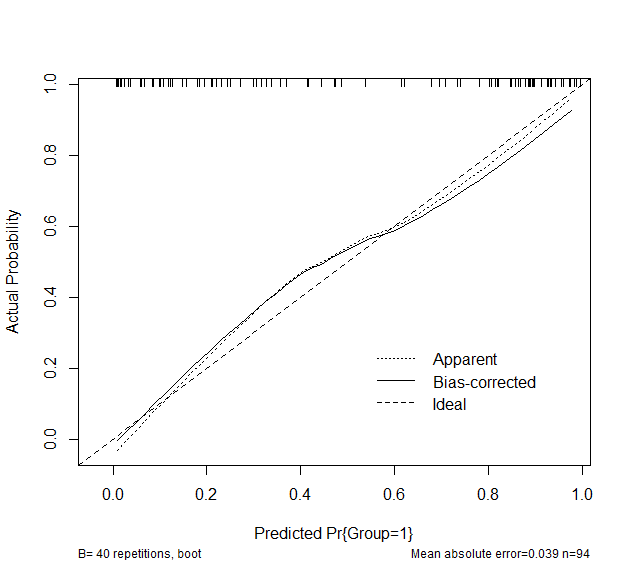


**Supplementary Table 4**

| **effect** | **ORu** | **CIu** | **Pvalueu** | **ORm** | **CIm** | **Pvaluem** |
| --- | --- | --- | --- | --- | --- | --- |
| Albumin | 1.212 | 1.009~1.456 | 0.040 | 1.244 | 1.013~1.526 | 0.037 |
| AngII | 1.040 | 1.001~1.080 | 0.046 | 1.056 | 1.007~1.107 | 0.025 |
| Weight | 1.098 | 1.041~1.158 | 0.001 | 1.100 | 1.039~1.166 | 0.001 |

（1）Hosmer-Lemeshow test：chisq=5.261, df=9, P= 0.811

（2）Calibration plot:


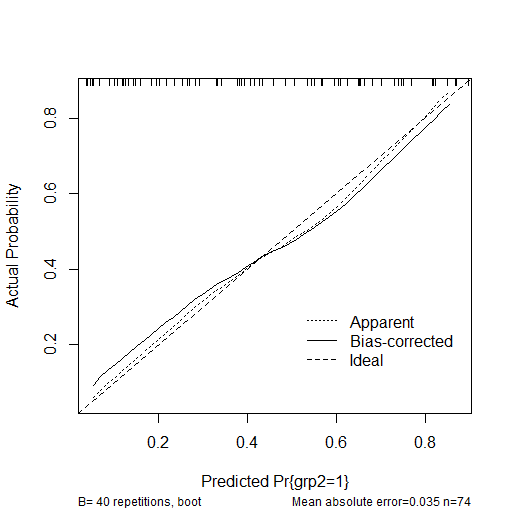

Supplement: Supplementary Materials — We performed the Hosmer-Lemeshow test and provided a calibration plot for the logistic model to confirm that our logistic model in Table 2 is reliable. We also performed the Hosmer-Lemeshow test and provided a calibration plot for the logistic model to confirm that our logistic model in Table 4 is reliable. [file 5647161.f1.docx]
